# Author Correction: Solution-processed perovskite light emitting diodes with efficiency exceeding 15% through additive-controlled nanostructure tailoring

**DOI:** 10.1038/s41467-019-09013-3

**Published:** 2019-02-22

**Authors:** Muyang Ban, Yatao Zou, Jasmine P. H. Rivett, Yingguo Yang, Tudor H. Thomas, Yeshu Tan, Tao Song, Xingyu Gao, Dan Credgington, Felix Deschler, Henning Sirringhaus, Baoquan Sun

**Affiliations:** 1 Jiangsu Key Laboratory for Carbon-Based Functional Materials & Devices, Institute of Functional Nano & Soft Materials (FUNSOM), Joint International Research Laboratory of Carbon-Based Functional Materials and Devices, Soochow University, 199 Ren’ai Road, Suzhou, 215123 People’s Republic of China; 2 Cavendish Laboratory, Department of Physics, University of Cambridge, JJ Thomson Avenue, Cambridge, CB3 0HE UK; 3 Shanghai Synchrotron Radiation Facility (SSRF), Shanghai Institute of Applied Physics, Chinese Academy of Sciences, 239 Zhangheng Road, Pudong New Area, Shanghai, 201204 China

Correction to: *Nature Communications* 10.1038/s41467-018-06425-5; published online 24 September 2018

The original version of this Article contained an error in the spelling of the author Dan Credgington, which was incorrectly given as Dan Credington. This has now been corrected in both the PDF and HTML versions of the Article.

