# Supplementary material for: Solution-processed perovskite light emitting diodes with efficiency exceeding 15% through additive-controlled nanostructure tailoring
Source: Nat Commun. 2018 Sep 24;9:3892. doi: 10.1038/s41467-018-06425-5 (PMC6155305; doi:10.1038/s41467-018-06425-5)
Supplement: Supplementary file 1 — Supplementary Information [file 41467_2018_6425_MOESM1_ESM.pdf]

Supplementary Information for

Solution-Processed Perovskite Light Emitting Diodes with Efficiency Exceeding 15% through Additive-Controlled Nanostructure Tailoring

Ban et al.

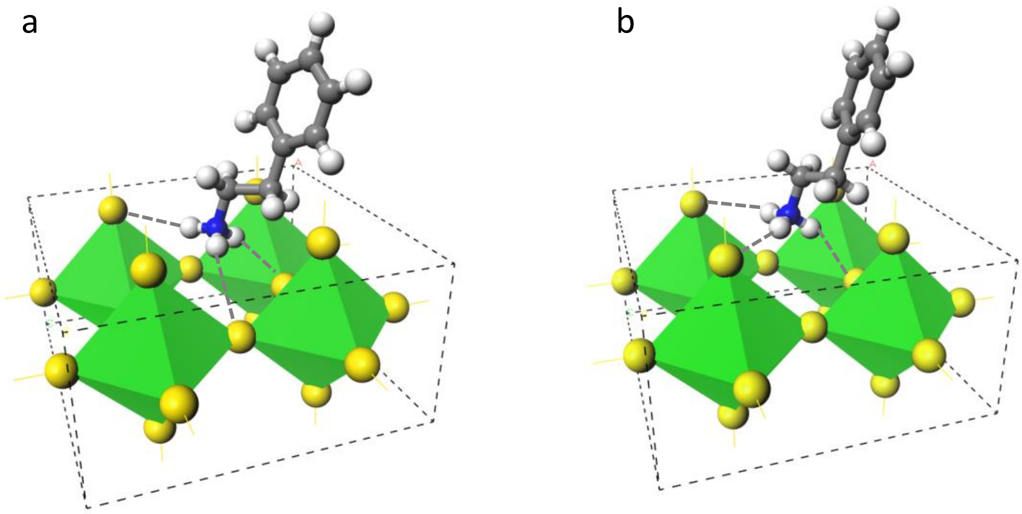

Supplementary Figure 1 Two hydrogen-bonding schemes for PEABr: (a) bridging halide configuration and (b) terminal halide configuration between PEABr and perovskite cubic structure.

a

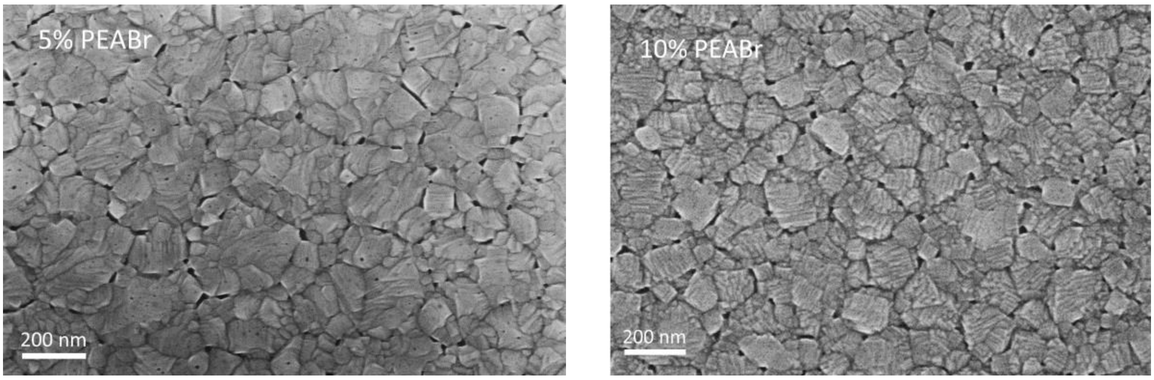

Supplementary Figure 2 SEM images of (a) 5% PEABr and (b) 10% PEABr perovskite film.

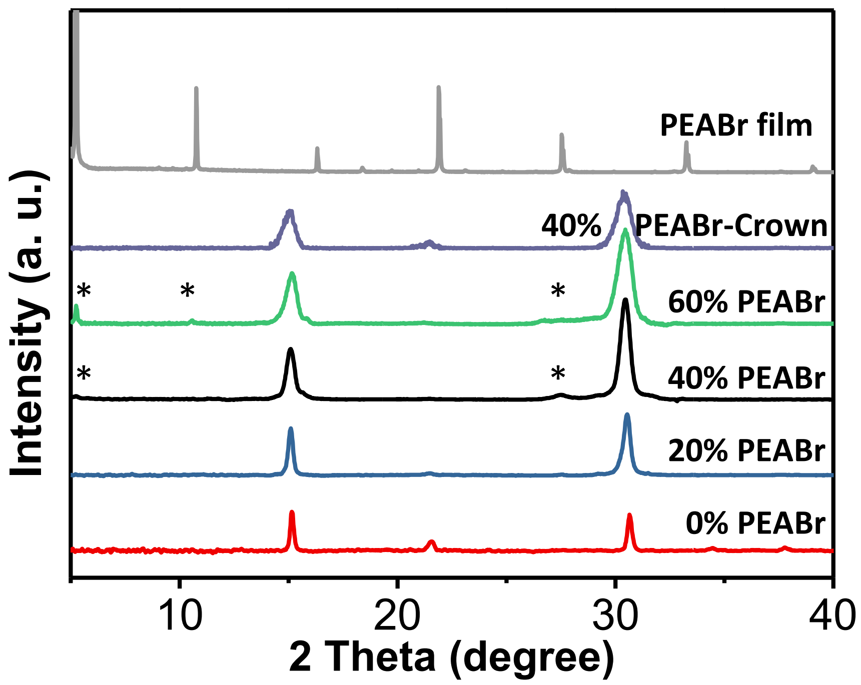

Supplementary Figure 3 θ-2θ XRD pattern of the perovskite films without, with different PEABr ratios, 40% PEABr-crown, and 0% PEABr film. '*' label indicates the PEABr diffraction peak.

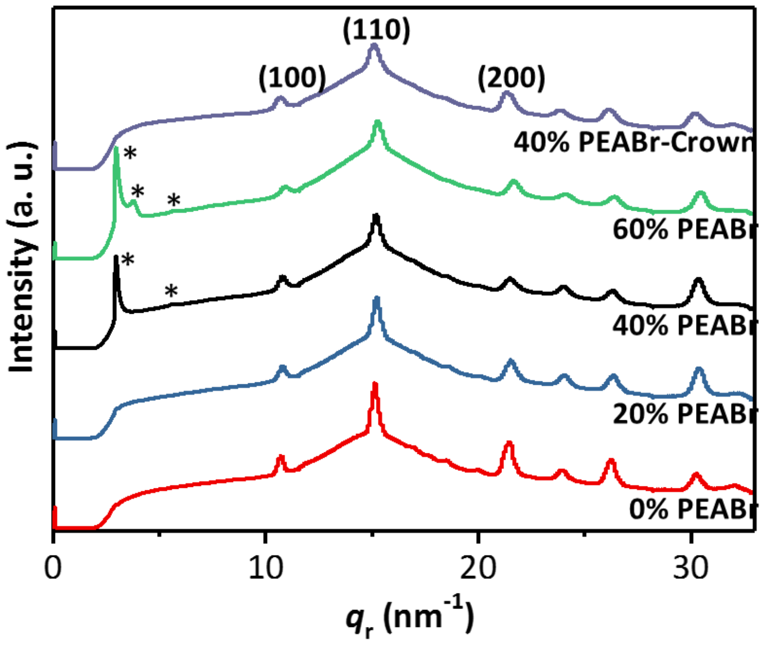

Supplementary Figure 4 Azimuthal integration (plotted) of GIXRD patterns in the perovskite films without, with different PEABr ratios, and 40% PEABr-crown. '*' label indicates the PEABr diffraction peak.

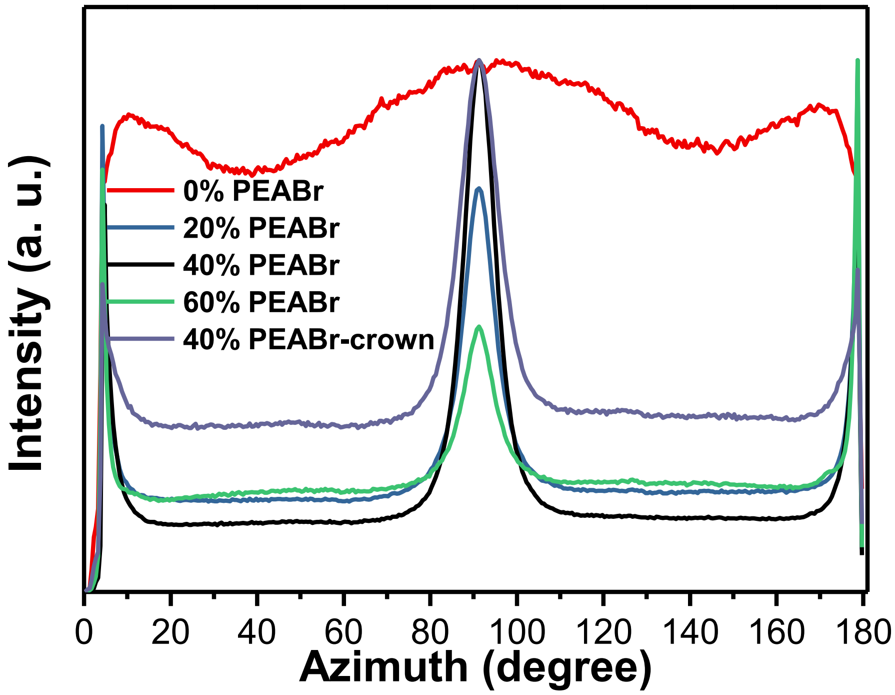

Supplementary Figure 5 Azimuthally integrated scattering intensity of different GIXRD profiles along the ring at q=10.7 nm^{-1} (100 plate) for the perovskite films with different PEABr ratio.

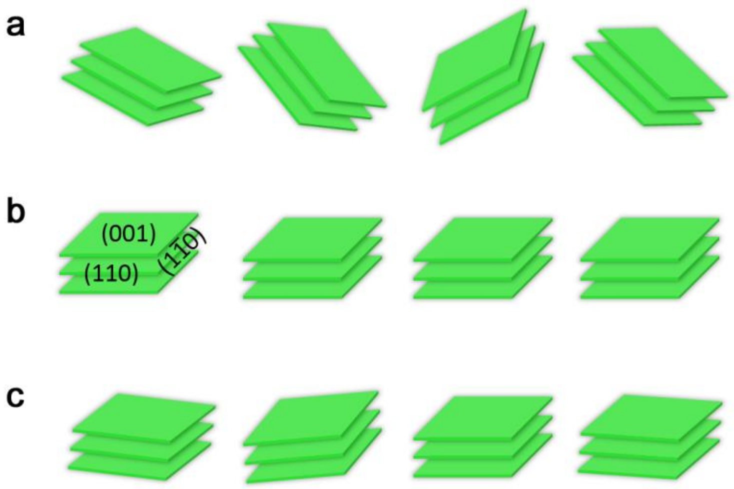

Supplementary Figure 6 Cartoon images of (001) plane orientation for the perovskite films (a) 0% PEABr, (b) 40% PEABr, and (c) 40% PEABr-crown. The other two planes of (110) and (1$\bar{1}$0) are also labeled in (b).

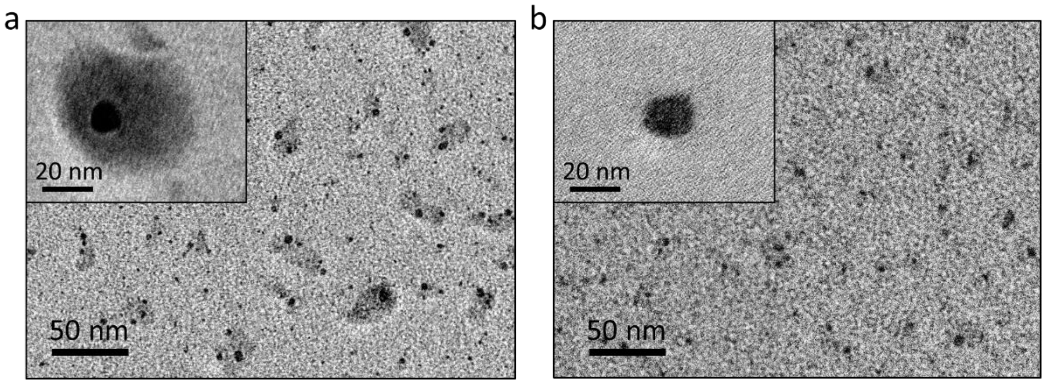

Supplementary Figure 7 TEM images for drop-cast 40% PEABr perovskite precursor solution (a) without and (b) with crown on copper grids coated with carbon film. Inset is TEM image with small scale bar.

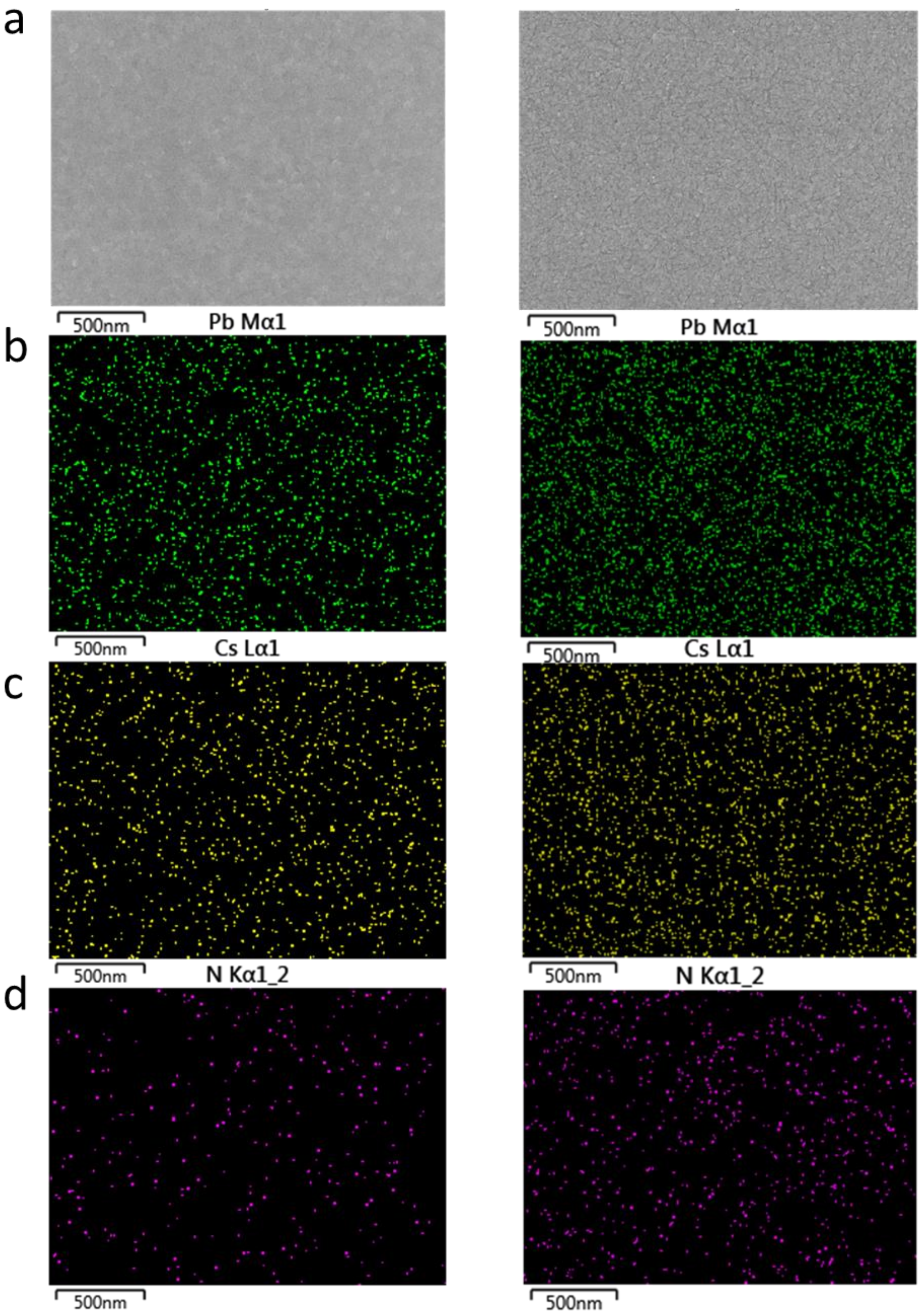

Supplementary Figure 8. SEM images of 40% PEABr perovskite without (left) and with (right) crown. SEM-EDX mapping the element distributions of (b) Pb, (c) Cs and (d) N in perovskite layer (40% PEABr, left; 40% PEABr-crown, right).

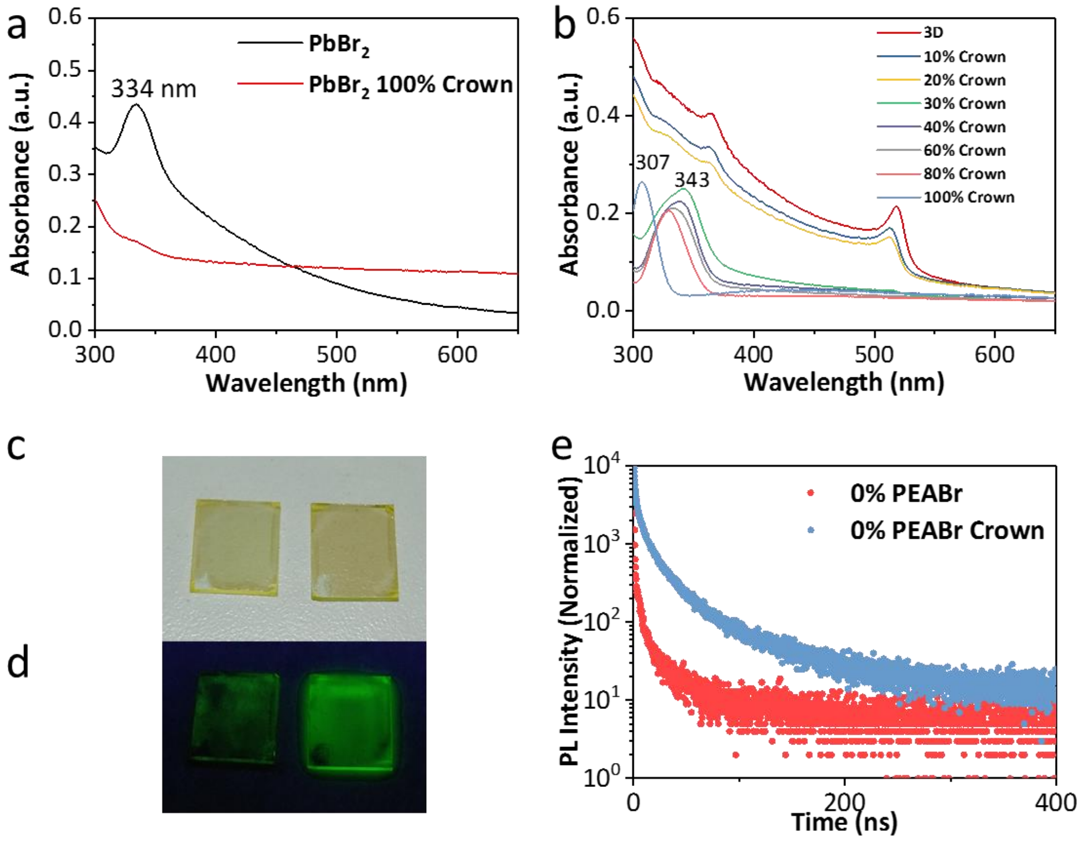

Supplementary Figure 9 (a) Absorption spectra of $PbBr_2$ film with crown ($PbBr_2$/crown=1:1, mole ratio) and without crown. (b) Absorption spectra of the films made from CsBr and $PbBr_2$ precursor solution with different amount of crown. Mole ratios are from 0 to 100%. (c) Photographs of 0% PEABr perovskite film without (left) and with (right) 7% crown (d) under ultraviolet lamp excitation (365 nm). (e) Time-dependence of PL intensity for 0% PEABr perovskite film without and with 7% crown.

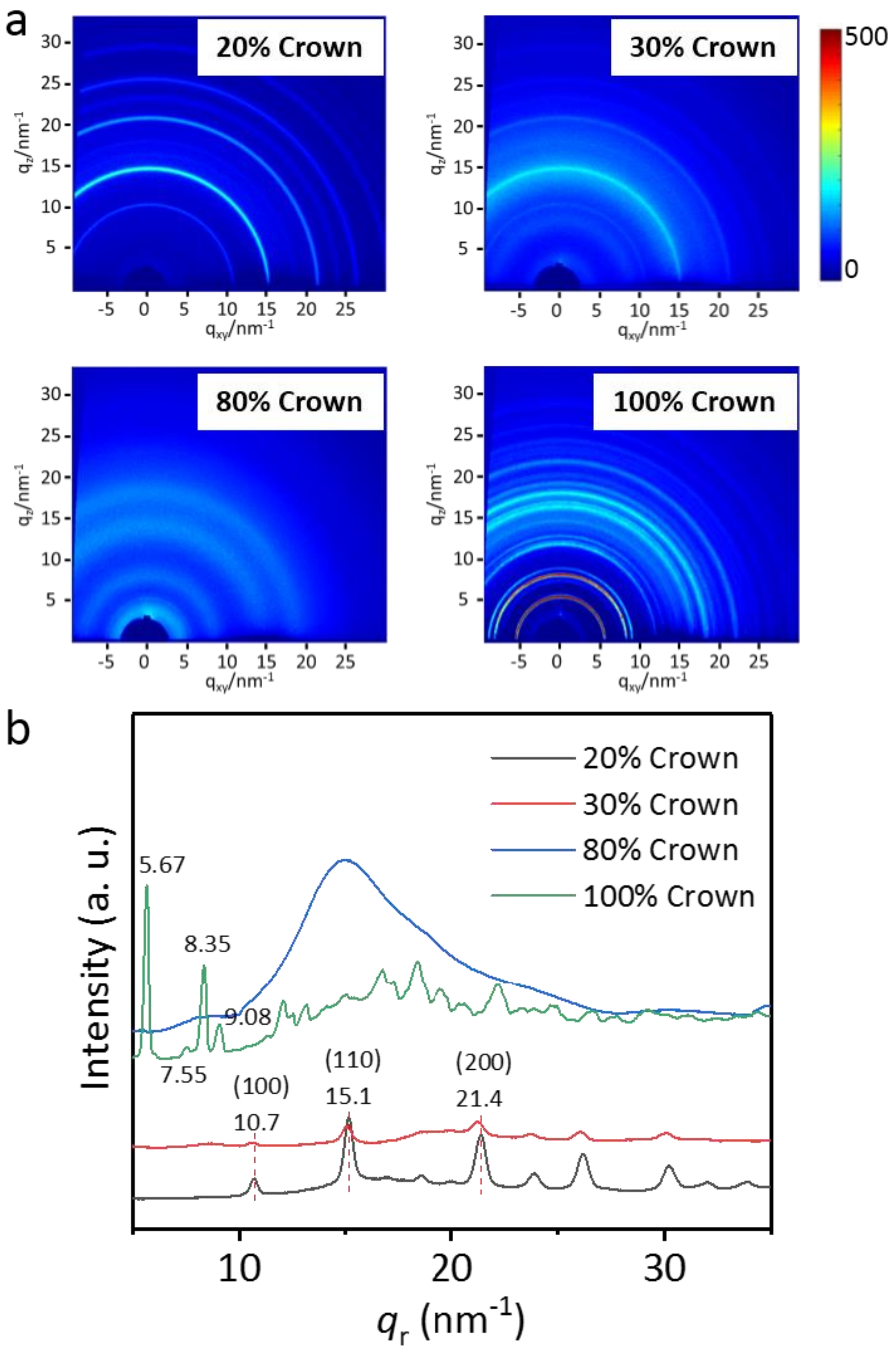

Supplementary Figure 10 (a) 2D GIXRD spectra of the films fabricated from CsBr and $PbBr_2$ precursor solution with different mole ratio of crown. The mole ration of crown/CsBr and $PbBr_2$=20%, 30%, 80% and 100% respectively. (b) Integrated 2D GIXRD diffraction signal from the 2D GIXRD spectra in (a).

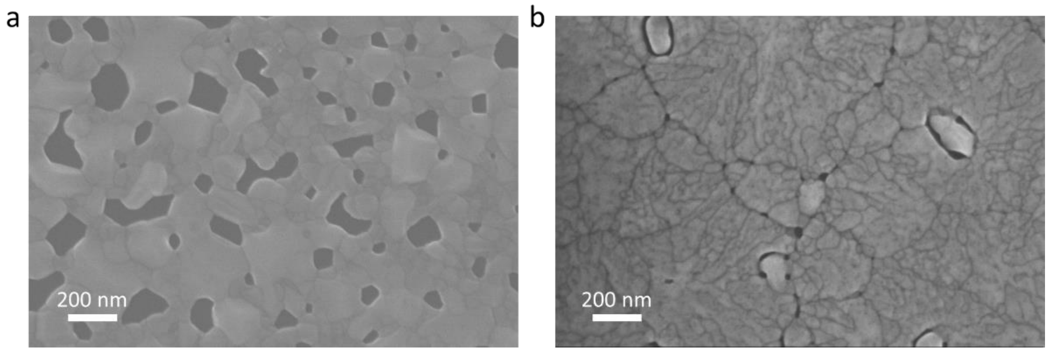

Supplementary Figure 11 SEM images of 0% PEABr $CsPbBr_3$ perovskite films (a) without and (b) with 7% mole ratio crown.

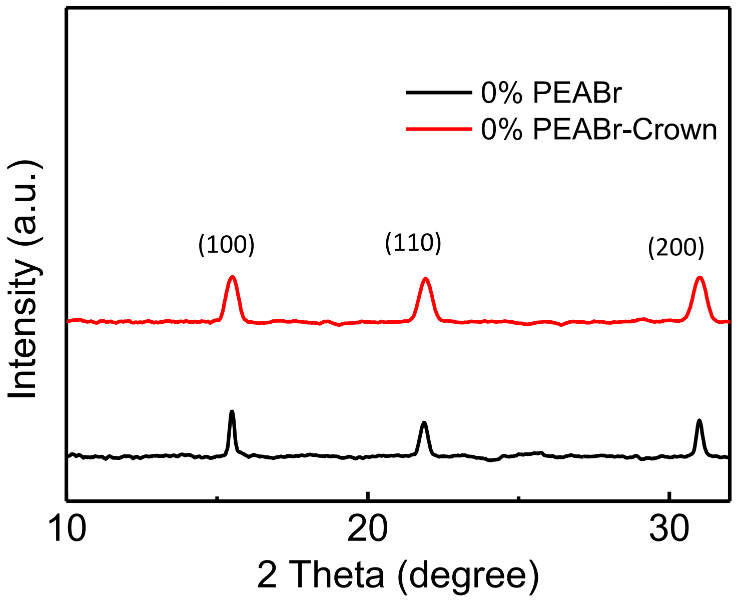

Supplementary Figure 12 θ-2θ XRD for 0% PEABr perovskite films with (7% mole ratio) and without crown.

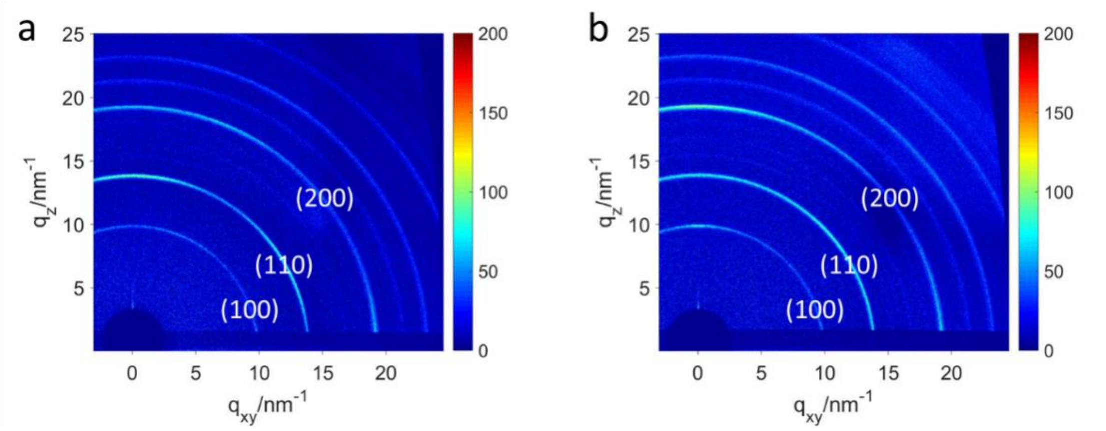

Supplementary Figure 13 2D GIXRD patterns for 0% PEABr $CsPbBr_3$ perovskite films (a) without and (b) with 7% mole ratio crown.

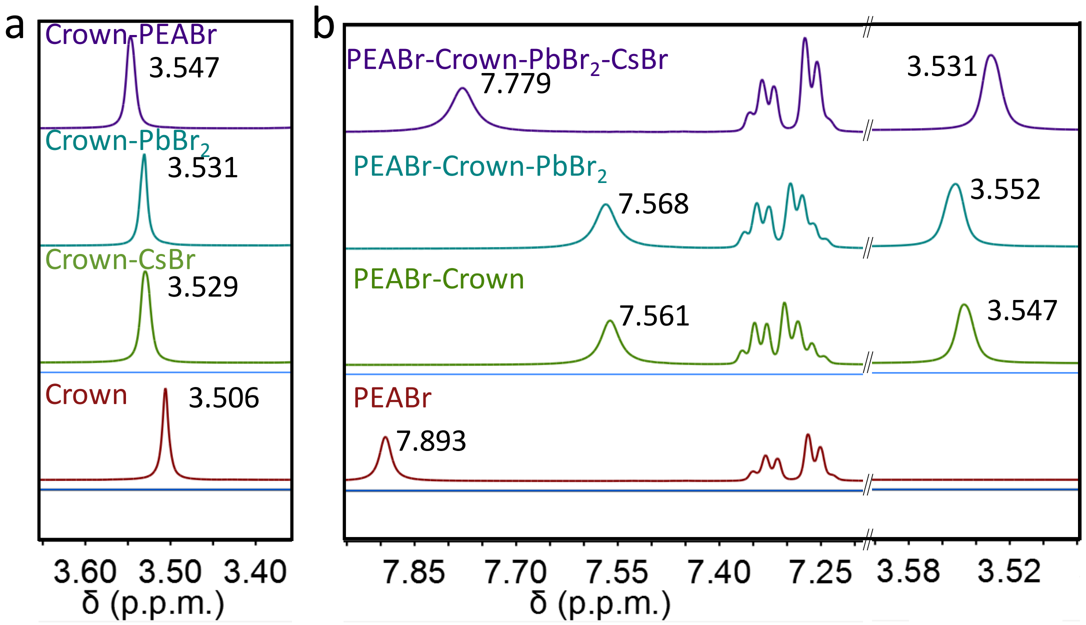

Supplementary Figure 14 ^{1}H NMR spectra in deuterated DMSO solution. (a) Proton resonance signals of crown in pristine crown solution, CsBr-crown solution: $PbBr_2$-crown and PEABr-crown solution, respectively. All the chemicals mole ratios are 1:1; (b) Proton resonance signals in PEABr-crown (mole ratio: 1:1) solution, crown-PEABr-$PbBr_2$ (mole ratio: 1:1:1) solution and crown-CsBr-PEABr-$PbBr_2$ (mole ratio: 1:1:1:1) solution, respectively.

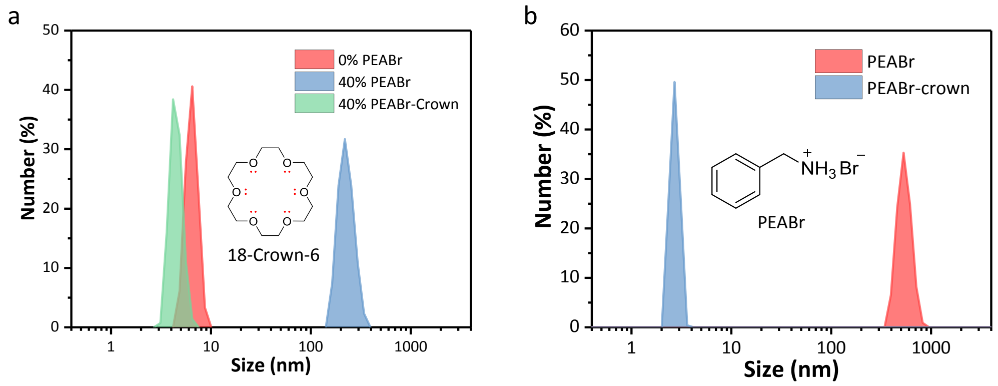

Supplementary Figure 15 (a) DLS measurements of different perovskite precursor solution of 0% PEABr (red), 40% PEABr (blue) and 40% PEABr-crown (gray) in DMSO. (b) DLS measurements of pristine PEABr (red) and mixture of PEABr and crown (blue) in DMSO. Inset images in (a) and (b) are the organic molecular structures of 18-crown and PEABr, respectively.

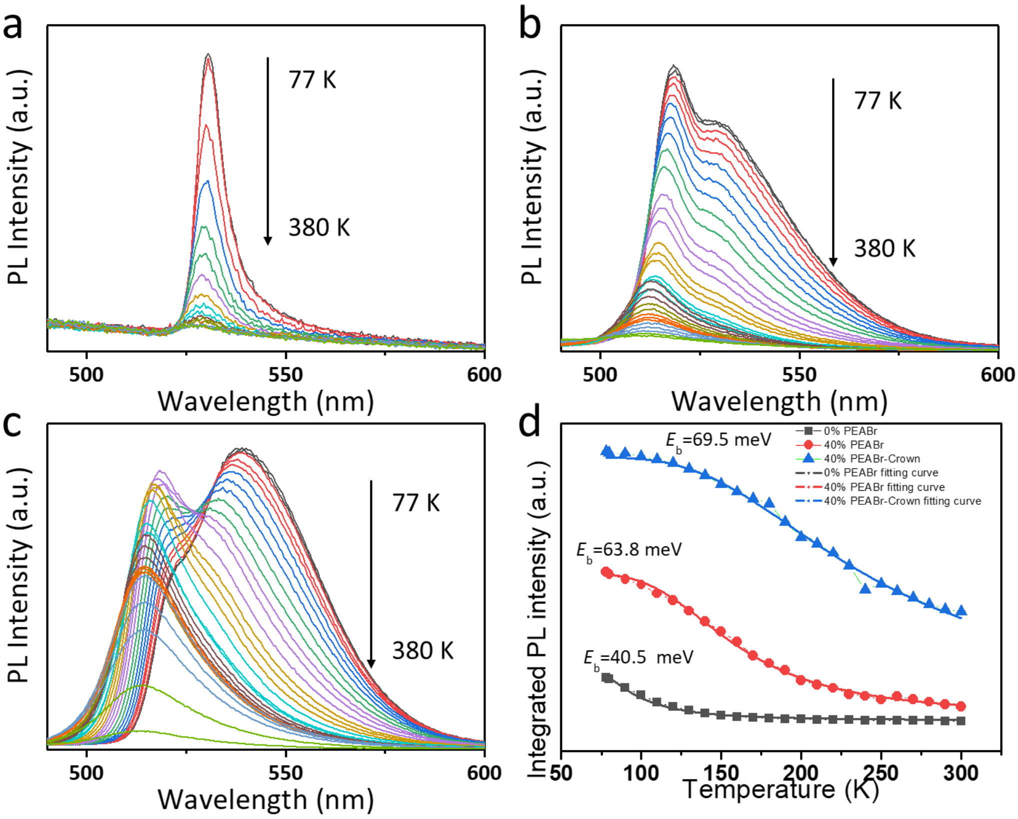

Supplementary Figure 16 Temperature-dependent PL of the different perovskite films (a) 0% PEABr, (b) 40% PEABr, and (c) 40% PEABr-crown. The first curve is measured at 78K. Then the other curves with a step of 10K from 90K to 380K. (d) Their corresponding temperature (T)-dependent integrating PL intensity (I(T)) against T and fitting curves (E_b is fitted by the equation that discussed above).

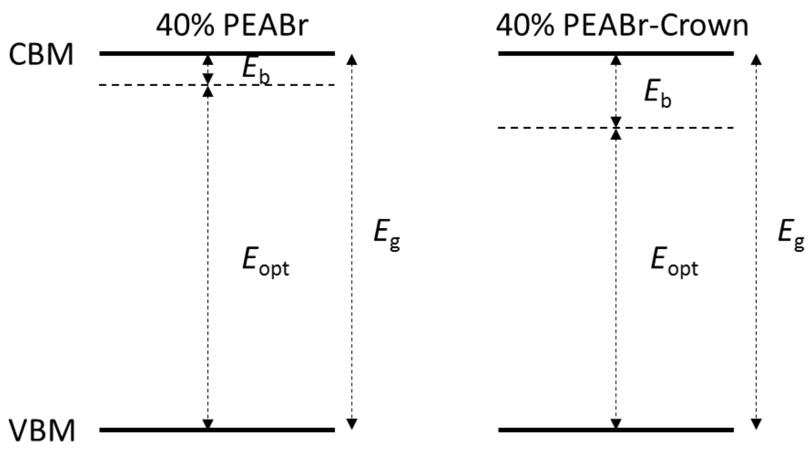

Supplementary Figure 17 Energy diagram schematically indicating the electronic bandgap (E_g), the optical bandgap (E_{opt}), and the exciton binding energy (E_b) of 40% PEABr with and without crown. CBM: conducting band maximum; VBM: valence band minimum.

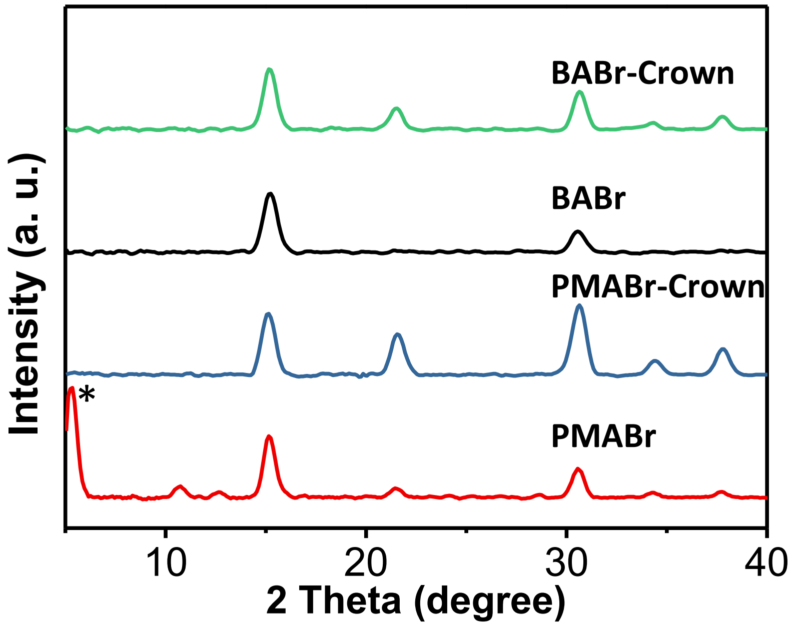

Supplementary Figure 18 θ-2θ XRD and PLQY value of perovskite film without and with 40% PMABr and BABr. '*' labelled peak shows PMA crystal diffraction.

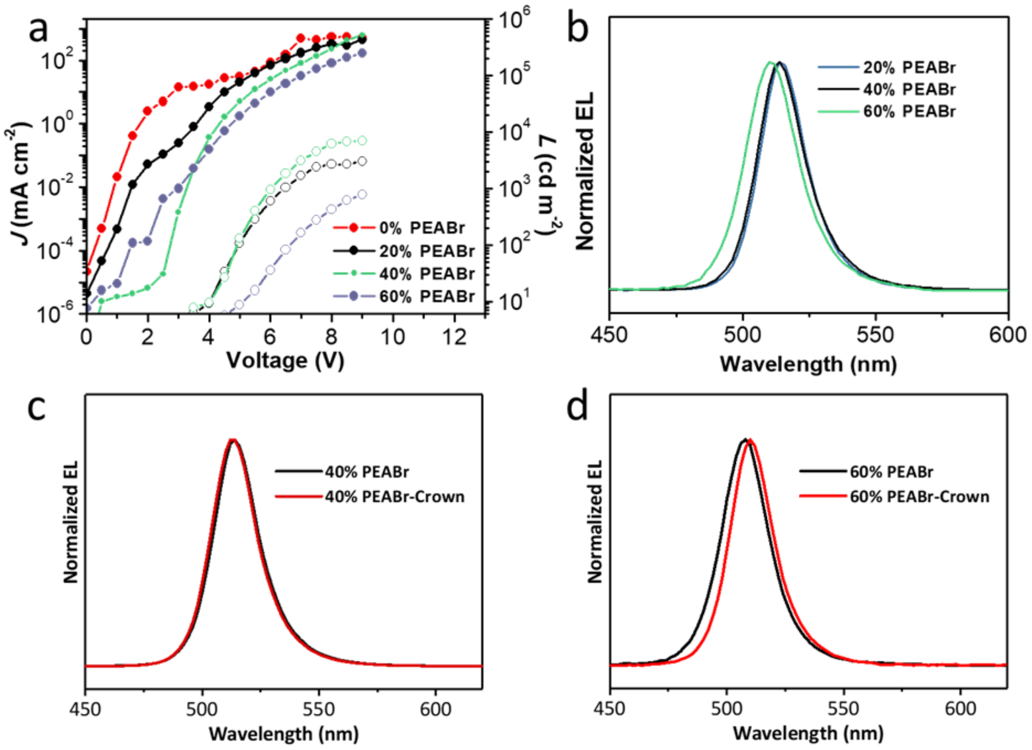

Supplementary Figure 19 (a) Current density-voltage-luminance (*J*–*V*–*L*) curves; (b) Normalized EL spectra of perovskite LEDs based on the perovskite films without and with different PEABr ratio; (c) Normalized EL spectra of perovskite LEDs based on the 40% PEABr perovskite films without and with crown; (d) Normalized EL spectra of perovskite LEDs based on the 60% PEABr perovskite films without and with crown.

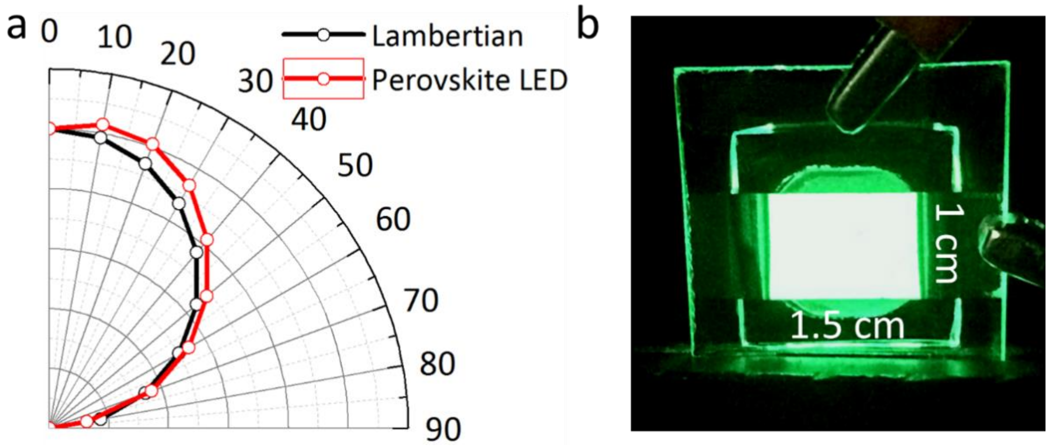

Supplementary Figure 20 (a) Angular intensity profile of a perovskite LED based on 40% PEABr-crown perovskite film compared with a Lambertian radiator emitting in normal and off normal direction. The angular dependent Lambertian emitter profile is calculated by an equation of $I = I_0 \cos\theta$. Here, I is light intensity at an angle of θ. I_0 is the light emitting light intensity at normal direction. Here, I_0 is assumed to be '1'. (b) A photograph of working 40% PEABr-crown LED device with an emitting size of 1.5 $\times$ 1 cm^2 operated at a bias of 4 V.

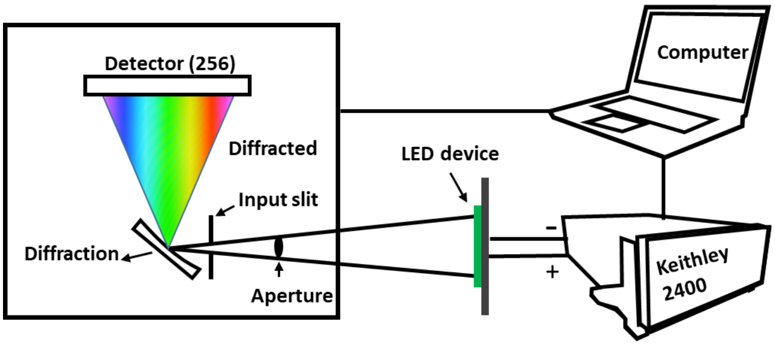

Supplementary Figure 21 Cartoon image of LED device characterization system. Current density–voltage characteristics were driven and collected by Keithley 2400, while PhotoReasearch Spectrometer PR670 was used for light output measurements.

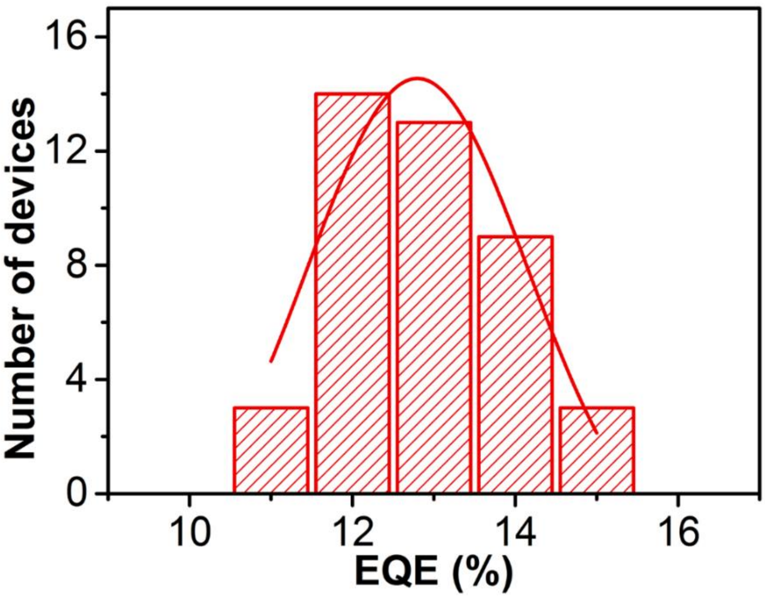

Supplementary Figure 22 EQE histogram of 42 LED devices for perovskite LEDs based on 40% PEABr-crown perovskite film. The fitting curve is a distribution function, demonstrating the spread in measured performance.

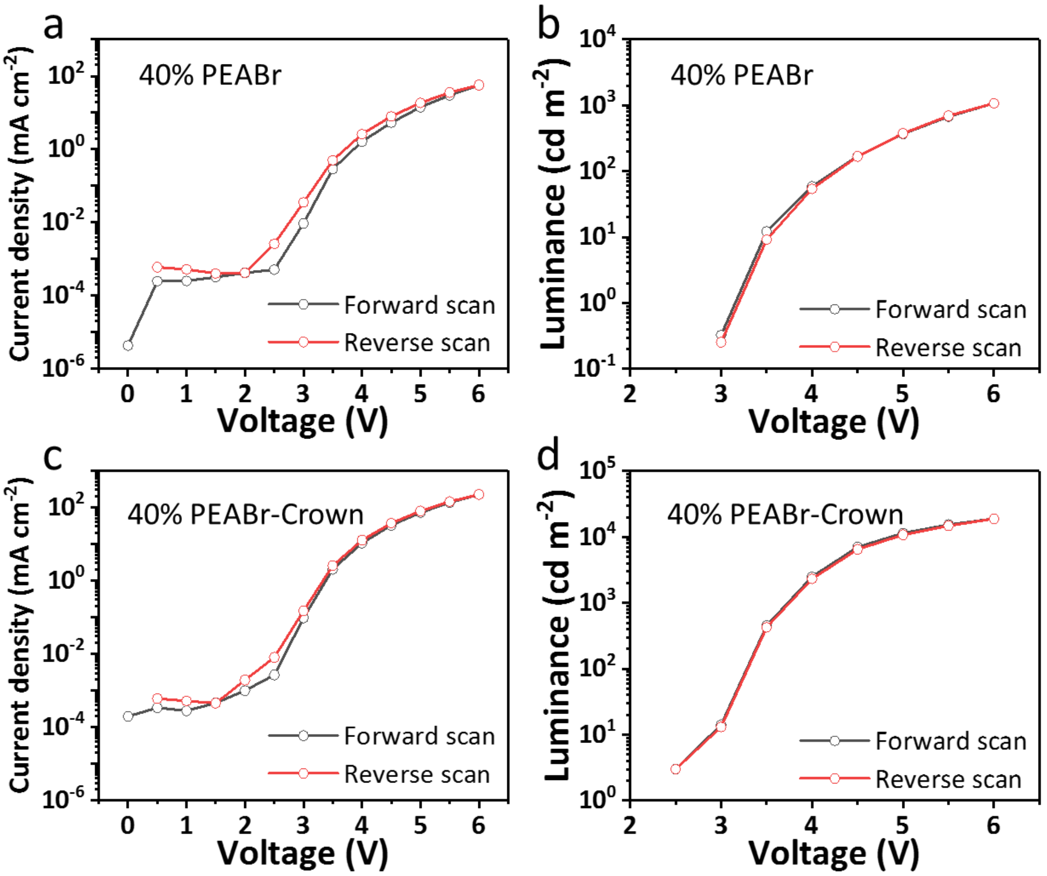

Supplementary Figure 23 (a) *J*-*V* curve and (b) *V*-*L* curve of 40% PEABr perovskite LED with forward and backward scanning, (cd) *J*-*V* curve and (d) *V*-*L* curve of 40% PEABr-crown perovskite LED with forward and backward scanning.

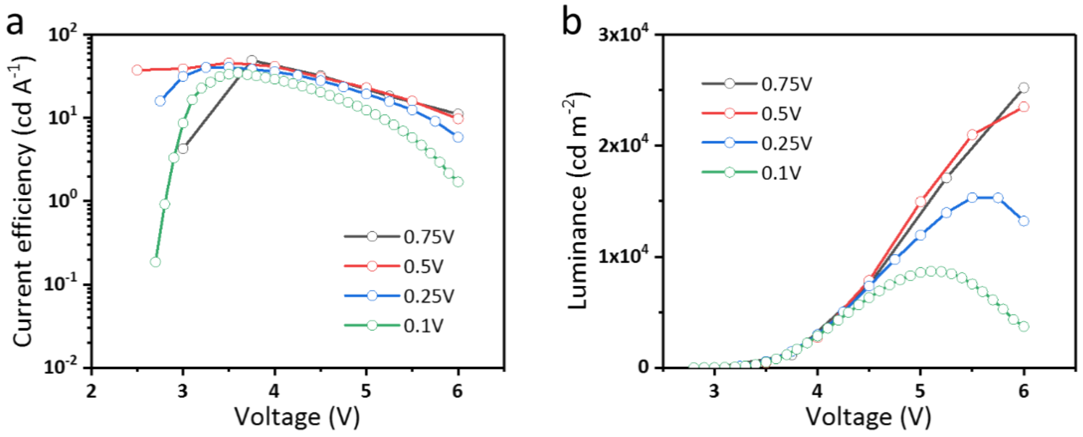

Supplementary Figure 24 (a) Scanning rate dependent current efficiency and (b) *V-L* curves for 40% PEABr-crown perovskite LEDs. The scan rate of 0.75, 0.5, 0.25, 0.1 V step is around 0.15, 0.1, 0.05 and 0.02 V s^{-1}, respectively.

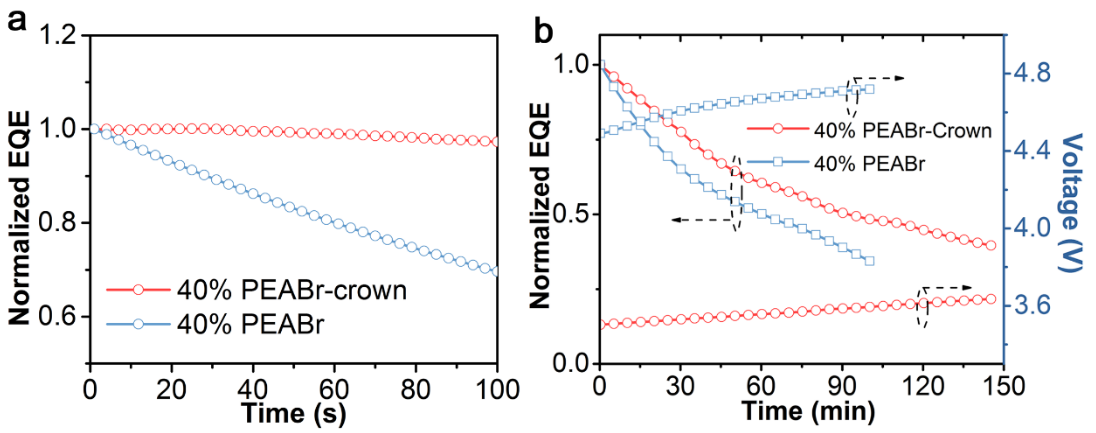

Supplementary Figure 25 Steady-state EQE measurement for perovskite LEDs based on the 40% PEABr perovskite films without and with crown (a) The devices were driven by a voltage of 3.5 V for a short period; (b) The devices were driven by a current level of 2 mA cm^{-2} for a long period.

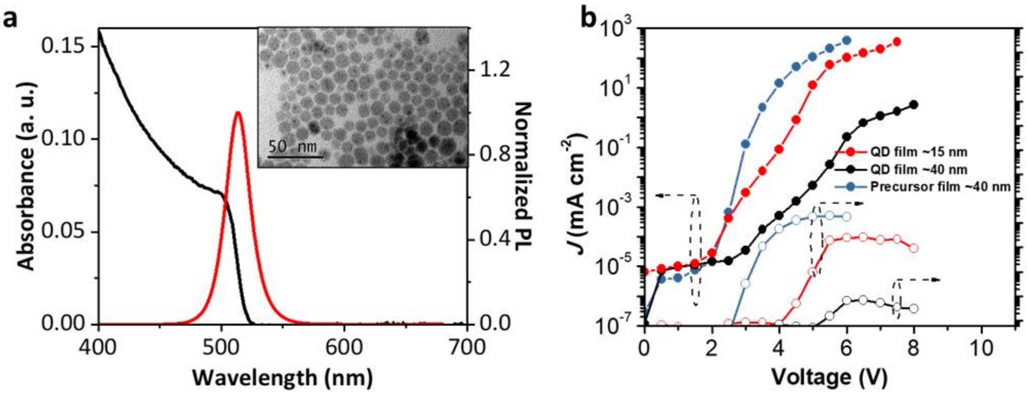

Supplementary Figure 26 (a) UV-Vis absorption and PL of colloidal $CsPbBr_3$ nanocrystal (TEM image inset), (b) *J*–*V*–*L* curves of colloidal $CsPbBr_3$ nanocrystals based LEDs with different thickness and precursor (40% PEABr with crown) based LEDs.

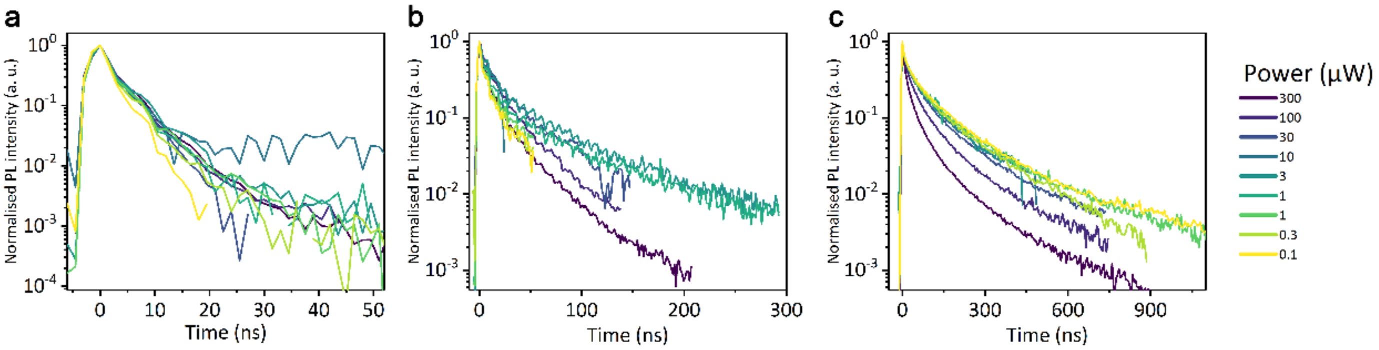

Supplementary Figure 27 PL intensity with different optical excitation density against time for the perovskite films without and with inorganic ligand. (a) 0% PEABr, (b) 40% PEABr, and (c) 40% PEABr-crown.

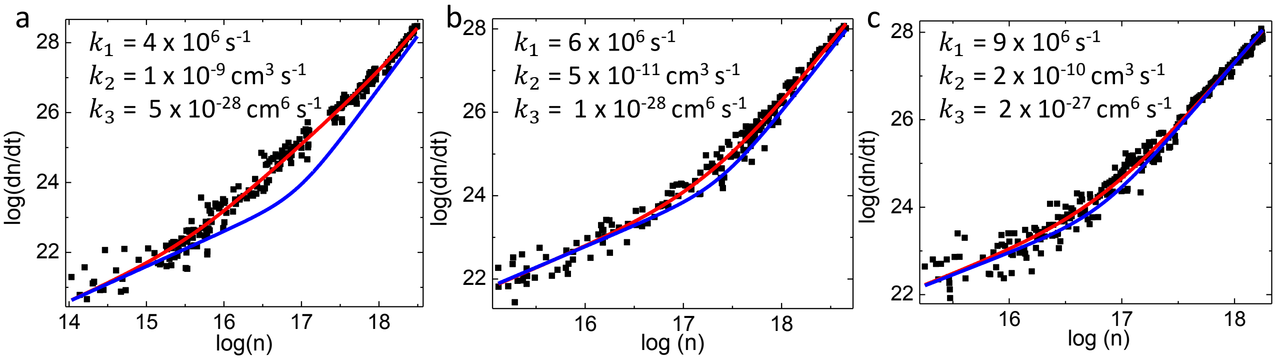

Supplementary Figure 28 Fits to the experimental data presented in Figure 4d-f for (a) 0% PEABr, (b) 40% PEABr, and (c) 40% PEABr-crown, using the function: $\log\left(-\frac{\mathrm{d}n}{\mathrm{d}t}\right) = \log\left(k_1 \cdot 10^{\log(n)} + k_2 \cdot 10^{2\log(n)} + k_3 \cdot 10^{3\log(n)}\right)$, which is equivalent to Supplementary Equation 1. Red lines show fits with k_1, k_2, k_3 unconstrained. Blue lines show the effect of subsequently setting k_2 to zero.

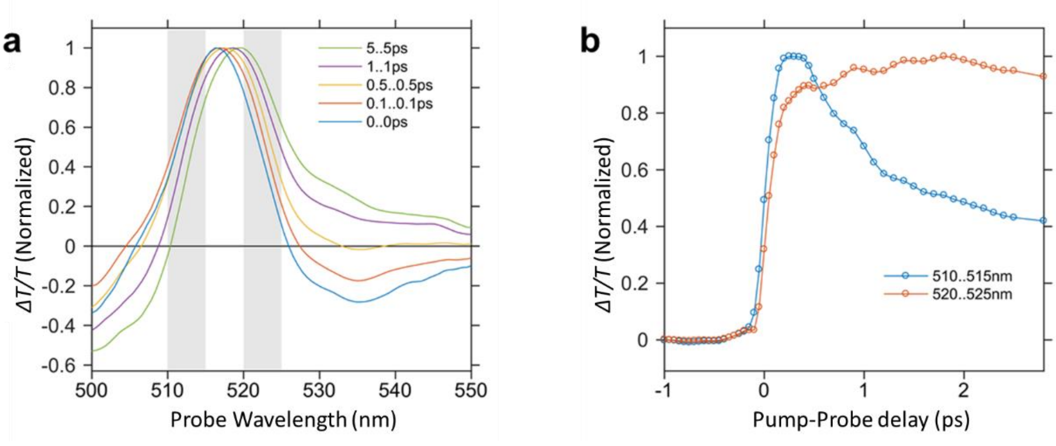

Supplementary Figure 29 (a) Transient absorption spectra and (b) time-delays and spectral regions for thin films of $CsPbBr_3$ for time-delays and spectral regions as indicated. Spectra show a small red-shift due to carrier cooling. No clear signatures of spectral relaxation and energy funnelling are seen in the kinetics for spectral regions above or below the main bleach peak.

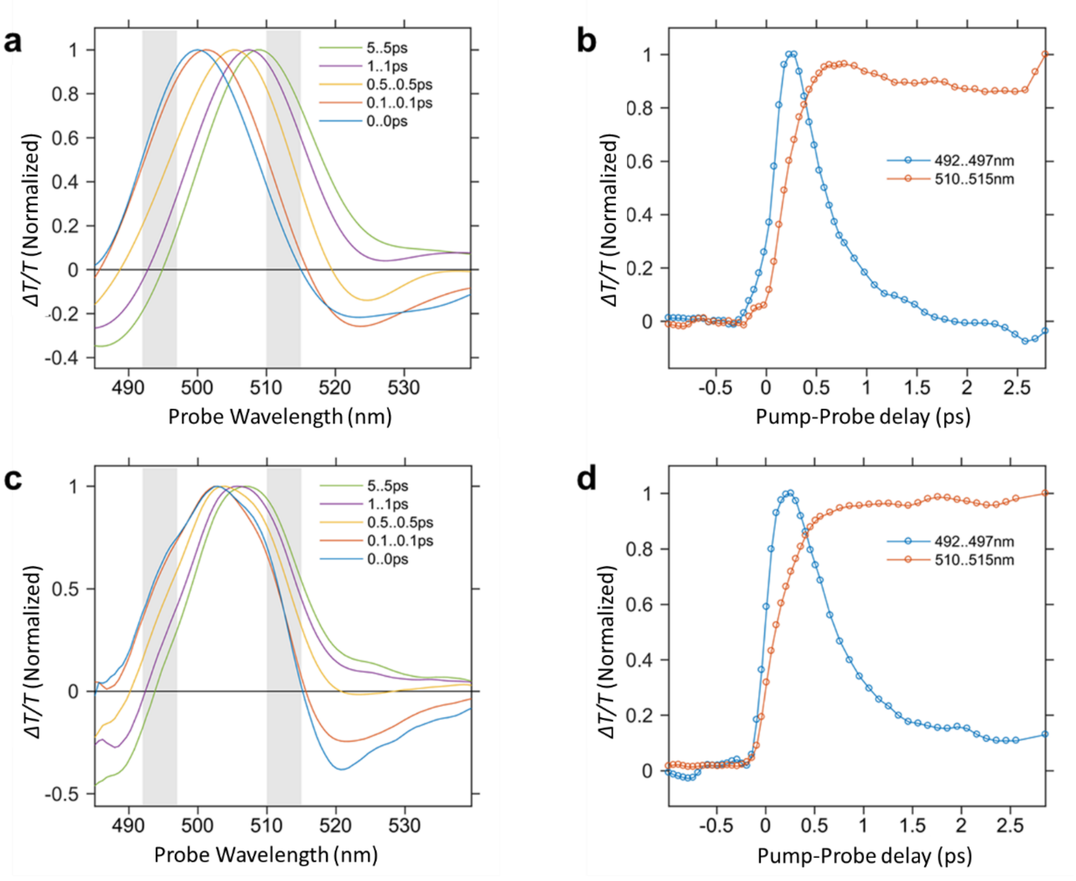

Supplementary Figure 30 Normalized transient absorption spectra and kinetics for thin films of (a) 40% PEABr perovskite and (c) 40% PEABr-crown perovskite, (b, d) kinetics of the transient absorption signal in two separate spectral regions as indicated in (a) and (c), respectively. Spectra show a strong red-shift due to energy funnelling. Signatures of energy funnelling are seen in the kinetics for spectral regions above or below the main bleach peak as full transfer of the signal intensity from high to low energy regions within 2 ps for both samples.

Supplementary Table 1 Average crystallite size of perovskite films extracted Scherer equation by θ-2θ XRD in Supplementary Figure 3.

**Sample**	**0% PEABr**	**20% PEABr**	**40% PEABr**	**60% PEABr**	**40% PEABr-Crown**
Average Size (nm)	42.8	33.9	18.5	14.3	13.3

Supplementary Table 2 PLQY of the 40% PMABr or BABr perovskite films without and with crown.

Ligand	PMABr	BABr
without	0.91	/
With Crown	21.6	12.6

Supplementary Table 3. A summary for published output characteristics of representative perovskite LEDs.

Perovskite Type	Device	EL Peak [nm]	EQE [%]	CE [cd A^{-1}]	L_{max} [cd m^{-2}]	V_t [V]	Publication Date [year.month]
$CH_3NH_3PbBr_3$	ITO/PEDOT:PSS/ $CH_3NH_3PbBr_3$/F8/Ca/ Ag	517	0.1		364	3.3	2014.08[1]
$CH_3NH_3PbBr_3$	Glass/SOCP/ $CH_3NH_3PbBr_3$/TPBi/ LiF/Al		8.53	42.9			2015.09[2]
$(C_6H_5C_2H_4NH_3)_2(CH_3NH_3)_{n-1}Pb_nI_{3n+1}$	ITO/TiO_2/2D-Perovskite/F8/MoO_3/Au		8.8			3.8	2016.06[3]
$(PEOA)_2(MA)_{n-1}Pb_nBr_{3n+1}$	ITO/PEDOT:PSS/ Perovskite/TPBi/Ba/ Al	506/ 520	2.84	8.23	64.2		2016.09[4]
$NMA_2FA_{n-1}Pb_nI_{3n+1}$	ITO/ZnO/PEIE/MQW /TFB/MoO_x/Au	763	11.7			1.3	2016.12[5]
BABr:$MAPbBr_3$/BAI :$MAPbI_3$	ITO/PVK/Perovskite/ TPBi/LiF/Al	513/ 748	9.3/ 10.4	17.1/ 0.09			2017.02[6]
$NMA_2(FA/Cs)_{n-1}Pb_nI_{3n+1}$	ITO/ZnO/PEIE/MQW /TFB/MoO_x/Au	688	3.70		440	2.0	2017.03[7]
$(PEA)_2(MA)_{n-1}Pb_nBr_{3n+1}$	ITO/PEDOT:PSS/ Perovskite/TPBi/LiF/ Al		7.4		8400		2017.05[8]
$Cs_{0.87}MA_{0.13}PbBr_3$	ITO/ZnO/PVP/ Perovskite/CBP/ MoO_3/Al	520	10.43	33.9	91000	2.9	2017.06[9]
$PEA_2(FAPbBr_3)_{n-1}PbBr_4$	ITO/m-PEDOT:PSS/ Perovskite/TOTO/TP Bi/LiF/ Al	532	14.36	62.4	9120		2018.02.08[10]
PEABr:$CsPbBr_3$	ITO/poly-TPD/Perovskite/TPBi/LiF/Al	514	15.5	49.1	19540	2.8	Our work

Supplementary Table 4. A summary for published operational lifetime of perovskite LEDs

Perovskite Type	Device Structure	Operation Condition	Operation Lifetime	Publication Date [year.month]
$MAPbBr_3$	ITO/TiO_2/EA/MHP/SPB-02T/MoO_3/Au	L_0≈120 cd m^{-2}, at 4.8 V	L_{50}≈55 h	2015/05[11]
$CsPbBr_3$ NPs	ITO/PEDOT:PSS/ poly-TPD/PFI/MHP/TPBi/LiF/Al	At 5 V	L_{50}≈10 min	2016/01[12]
$CsPbBr_3$:CsBr = 1:0.4 (molar ratio)	ITO/PEDOT:PSS/MHP/ B3PYMPM/Cs_2CO_3/Al	L_0≈100 cd m^{-2}, at 66.67 mA cm-2	$L > L_0$ for >15 h	2016/09[13]
$CsPbBr_3$:PEO:PVP = 100:50:5 (weight ratio)	ITO/MHP/In:Ga	L_0≈100 cd m^{-2}, at 2.7 V	$L > L_0$ for >1 h	2016/09[14]
NMAI: FAI:PbI_2 = 2:1:2 (molar ratio)	ITO/ZnO/PEIE/MHP/TFB/ MoO_x/Au	At 10 mA cm^{-2}	L_{50}≈100 min	2016/09[5]
$CsPbBr_3$ NPs	Au/p-MgNiO/MHP/PMMA/ n-MgZnO/n+-GaN	At 10 V, un-encapsulated, under ambient condition (RH = 30–50%)	L_{80}≈9 h	2016/12[15]
BAI:$MAPbI_3$ = 20:100 (near infrared (NIR))	ITO/poly-TPD/ MHP/TPBi/LiF/Al	At 3 mA cm^{-2}	L_{70}≈300 min (NIR)	2017/01[16]
BABr:$MAPbBr_3$ = 20:100 (green)	ITO/PVK/ MHP/TPBi/LiF/Al	At 3 mA cm^{-2}	L_{50}≈48 min (green)	2017/01[16]
$MAPbBr_3$	ITO/PEDOT:PSS/MHP/TPBi/LiF/Al	At 6.5 V	L_{50}≈100 s	2017/03[17]
$FA_{0.8}Cs_{0.2}PbBr_3$ NPs	ITO/PEDOT:PSS/TFB/MHP/ TPBi/LiF/Al	Not reported	L_{50}≈80 s	2017/03[18]
$MAPbI_3$	ITO/poly-TPD/MHP/TPBi/ LiF/Al	At 3 mA cm^{-2}	EQE > EQE_0 for >300 min	2017/04[19]
NMAI:CsCl:PbI_2 = 2:1:2 (molar ratio) (red, 688 nm)	ITO/ZnO/PEIE/MHP/TFB/ MoO_x/Au	At 10 mA cm^{-2}	L_{50}≈5 h	2017/04[7]
$CsPbBr_3$:PEO = 6:1 (weight ratio)	ITO/PEDOT:PSS/MHP/TPBi/LiF/Al	L_0≈1000 cd m^{-2}	L_{80}≈80 h	2017/05[20]

$Cs_{0.87}MA_{0.13}PbBr_3$: PVP	ITO/ZnO/PVP/MHP/ CBP/MoO_3/Al	L_0≈ 65 cd m^{-2}, at 5 V	L_{50}≈1 h	2017/06[21]
$(BA)_2(MA)_2Pb4I_{13}$	ITO/PEDOT:PSS/MHP/ PCBM/Al	L_0≈6 W Sr^{-1} m^{-2}, At 2 V	More than 14 h	2017/10[22]
NMAI:FAl:PbI_2 = 2:1.9:2 (molar ratio)	ITO/ZnO/PEIE/MHP/TFB/ MoO_x/Au	At 100 mA cm^{-2}	L_{50}≈30 min	2018/02[23]
$PEA_2(FAPbBr_3)_{n-1}$ $PbBr_4$	ITO/m-PEDOT:PSS/MHP/ TOPO/TPBi/LiF/Al	At 0.5 mA cm^{-2}	L_{50}≈120 min	2018/02[10]
$PEA_2Cs_{n-1}PbnBr_{3n+1}$	ITO/poly-TPD/PFN/MHP/ TPBi/LiF/Al	At 2 mA cm^{-2}	L_{50}≈90 min	Our work

Supplementary Note

Supplementary Note 1 - Discussion of effect of PEABr on impeding perovskite crystal growth

The impeding effect of PEABr on perovskite crystal growth can be ascribed to the strong hydrogen bond between hydrogen atom of PEABr and halide atom in $PbBr_6$ (N-H ··X), which can occur in two forms: either bridging halide configuration (two bridging halides and one terminal halide) or terminal halide configuration (two terminal halides and one bridging halide)[24]. The configurations are determined by geometric constraints of the organic groups and organic tails (as shown Supplementary Figure 1). Since PEABr is much larger than cesium cation, the growth of perovskite crystallites (A=PEABr) is impeded due to the incompatibility between relative large PEABr ammonium ions and PbX_4 (X = I, Br, Cl) octahedral layers.

It is quite difficult to directly observe the OHP nanoplatelets when the PEABr ratio is over 40%. Fortunately, when the PEABr ratio is 5% or 10%, thick nanoplatelets can be observed by SEM (as shown Supplementary Figure 2). In these images, self-packed nanoplatelets are easily recognized.

Supplementary Note 2 - Determination of microstructure by XRD, TEM and scanning electron microcopy- energy-dispersive X-ray spectroscopy (SEM-EDX)

In θ-2θ XRD scans detecting Bragg peaks normal to the substrate (Supplementary Figure 3), the (110) plane peak at θ = 21.6 ° disappears while the (100) and (200) plane diffractions become stronger with addition of PEABr which reveals that the crystal (001) plane displays preferential orientation in the plane of the substrate. Supplementary Figure 4 shows the GIXRD profiles of perovskite films with different amount of PEABr. The diffraction spots or rings at q= 10.7, 15.2 and 21.5 nm^{-1} are assigned to (100), (110) and (200) planes of the perovskite structure, which is in line with the θ-2θ XRD measurement. According to normal XRD and GIXRD analysis, it is safely concluded that there is a preferential orientation along (001) plane direction in OHP films with adding PEABr.

We also plot the azimuthally integrated scattering intensity of different GIXRD profiles along the ring at q= 10.7 nm^{-1} for (100) plane in Supplementary Figure 5. Preferential orientations with obvious peaks at the azimuth angles of 90 ° are observed after incorporating PEABr into perovskite film. These results illustrate that an ordered crystal orientation is facilitated by the addition of PEABr, which reveals the preferential growth unit cell[3], as shown in Supplementary Figure 6. This preferential cell should be ascribed by self-packed $PEA_2Cs_{n-1}Pb_nBr_{3n+1}$ nanoplatelets, which is consistent with the later optical measurement. It worth noted that this preferential orientation become weak over 40% PEABr, however, the reason is not clear.

As shown in Supplementary Figure 3, besides the perovskite diffraction peaks, some unexpected small peaks at 5.26^o, 10.6^o and 27.9^o are also observed with increasing the

PEABr molar ratio. Interestingly, XRD pattern of pristine PEABr film also exhibits strong peak at 5.26^{o}, 10.7^{o} and 27.5^{o}, indicating that PEABr is also crystalized in OHP film formation process. Synchrotron based 2D GIXRD measurements confirmed this point. The PEABr diffraction signal from GIXRD at q= 2.97 nm^{-1} becomes stronger with the increased PEABr ratio, which is ascribed to increasing PEABr self-crystallization (Figure 1 c, d and Supplementary Figure 4).

The addition of crown suppresses the formation of PEABr aggregates as well as reduced preferential orientation of (100) plane. As shown in Supplementary Figure 3, the (110) plane peak can be detected again upon adding crown due to its reduced preferential orientation of crystallites. In addition, azimuthally integrated scattering intensity of different GIXRD profiles along the ring at q= 10.7 nm^{-1} (100 plane) reveals that there is a reduced preferential orientation, as shown Supplementary Figure 5. This crystal unit orientation changes from $CsPbBr_3$ (0% PEABr) to the OHP film with PEABr or PEABr-crown is illustrated in Supplementary Figure 6.

The addition of crown only slightly affects the thickness of perovskite films (0% PEABr: 34.56±0.58 nm, 0% PEABr-crown: 37.20±0.32 nm; 40% PEABr: 39.46±0.33 nm; 40% PEABr-crown: 42.65±0.15 nm; 60% PEABr: 45.13±0.46 nm; 60% PEABr-crown: 50.01±0.43 nm). It shows approximately 10% difference before and after crown addition. These relatively minor differences in thickness cannot explain the large differences of the PEABr diffraction signals in XRD and GIXRD.

TEM images for the 40% PEABr perovskite with and without crown further confirm the suppression of PEBr aggregation, as shown in Supplementary Figure 7. According

to these images, PEABr aggregation can be observed, and which may reveal that there is large phase segregation between PEABr and perovskite. After adding crown, the aggregation is significantly reduced and the crystallite size is uniform and small. Though drop-casting is different compared with spin-coating, the intrinsic properties should be same. According to TEM images, we can directly observe that there is a large phase separation between PEABr and perovskite.

In order to provide the further evidence for phase separation, SEM-EDX mapping is collected, as shown Supplementary Figure 8. After incorporating crown in 40% PEABr perovskite film, the distributions of Pb, Cs and N become more uniform than in the 40% PEABr perovskite film, which is evidence for less pronounced phase separation.

GIXRD measurement is also conducted for 0% PEABr $CsPbBr_3$ with different amount of crown, as shown in Supplementary Figure 10, which confirms the suppression of perovskite growth in the presence of crown. According to GIXRD images and integrated diffraction signal, the diffraction signal of 0% PEABr perovskite at 10.7, 15.1 and 21.4 nm^{-1} for (100), (110) and (200) planes become weaker with increasing crown mole ratio. When the crown ratio is 80%, the film becomes amorphous without any diffraction signal. When the crown ratio is 100%, some new diffraction peaks at 5.67, 8.35 and 9.08 nm^{-1} appear, which have not been identified yet.

Film morphology measurement is conducted for 0% PEABr $CsPbBr_3$ with and without crown. Here, the concentration of crown in optical measurement and LED

device is very small (only 3.5 mg ml^{-1}, mole ratio of crown/($CsBr+PbBr_2$) is 0.07). The restriction of perovskite growth can be identified. As shown SEM images of 0% PEABr $CsPbBr_3$ perovskite with and without 7% mole ratio crown in Supplementary Figure 11, pristine 0% PEABr perovskite film (0% PEABr) shows discontinuous and large crystallite size, while after adding small amount of crown, the crystallite size become smaller. According to SEM images (Supplementary Figure 11), the perovskite crystallite size is dramatically suppressed in the presence of crown. In addition, the film coverage ratio is also improved. The reduced crystallite size is in line with the blue-shift in PL and absorption spectrums (Figure 2a, b), indicating an enhanced spatial confinement.

θ-2θ XRD and GIXRD show no difference in diffraction peak position of 0% PEABr perovskite except crown ratio is over 30% (also observed from Supplementary Figure 10), meaning that the crystallite structure has no change besides the reduced crystallite size. θ-2θ XRD (Supplementary Figure 12) shows that the diffraction peak becomes wider, which further confirms the reduced crystallite size in the presence of 7% crown. The calculated crystallite size decrease form 45.8 nm to 20.3 nm according to Scherrer equation.

In conclusion, according to absorption, SEM, 2D GIXRD and θ-2θ XRD, the most important role of crown is suppressing PEABr self-packing aggregation and concomitant inhibiting of phase separation. Besides that, crown interacts with Pb^{2+} to suppress the perovskite growth. The amount of crown must be small enough to guarantee that not only PEABr aggregation can be suppressed, but also that it does not hinder perovskite growth significantly.

Supplementary Note 4 - ^{1}H nuclear magnetic resonance (NMR) spectra and Dynamic light scattering (DLS) measurements

To further confirm the interaction between crown and PEABr, CsBr, $PbBr_2$, we use ^{1}H NMR to characterize the interaction between crown and Pb^{2+}, Cs^{+}, PEA^{+}. ^{1}H NMR spectrum of crown with $PbBr_2$, CsBr, and PEABr individually are measured, respectively. The mole ratio between crown and $PbBr_2$ (CsBr, PEABr) is 1:1. As shown in Supplementary Figure 14a, the proton resonance signals of crown (peak at δ=3.506 p.p.m.) shift downfield when adding CsBr (3.529 p.p.m.), $PbBr_2$ (3.531 p.p.m.) and PEABr (3.547 p.p.m.), respectively. Here, such chemical downshift can be assigned to the hydrogen bond between PEA^{+} and oxygen atom in crown molecular, or coordinating bond between Pb^{2+}, Cs^{+} and crown molecular. These bond interactions result in proton chemical environmental change. And this downfield chemical shift for proton in crown increases from Cs^{+}, to Pb^{2+} and PEA^{+}, which reveals that the interaction between crown and PEA^{+} is the strongest.

This observed chemical shifts are consistent with the radius of Pb^{2+} (120 pm) being more suitable for crown (18-crown-6, hole radius is 130-160 pm) than Cs^{+} (169 pm), from which one would expect a stronger interaction between Pb^{2+} and 18-crown-6 than that between Cs^{+} and 18-crown-6. There are three hydrogen bonds between PEA^{+} and crown, so the interaction is expected to be the strongest among them. Besides that, the proton resonance signals of PEA^{+} (peak at δ=7.893 p.p.m.) shift towards upfield after incorporating with crown as shown in Supplementary Figure 14,

which can also be attributed to the hydrogen bonds discussed above.

In order to investigate the interaction in mixed system, we prepare two samples, PEABr-crown-$PbBr_2$ (molar ratio is 1:1:1) and PEABr-crown-$PbBr_2$-CsBr (molar ratio is 1:1:1:1). The ^1H NMR spectra for these two samples are shown in Supplementary Figure 14b. When there are crown, Pb^{2+} and PEA^+ in solution, the shifts of proton resonance signals of PEA^+ and crown is similar with PEA^+-crown sample. It means that PEA^+ and crown exhibit stronger interaction than Pb^{2+} and crown. However, when crown, Pb^{2+}, Cs^+, and PEA^+ are mixed together, the shifts becomes smaller. We speculate that it is ascribed to perovskite nanocrystal formation even in solution[25], which would reduce the interaction between crown and these three ions.

In summary, the interactions between crown and Pb^{2+}, Cs^+, PEA^+ are probed by ^1HNMR. The comparative strength of the interactions of crown is in an order of $PEA^+ > Pb^{2+} > Cs^+$.

To further confirm the interaction between crown and PEABr, precursors with and without crown in DMSO were measured by dynamic light scattering (DLS)[26], as shown in Supplementary Figure 15a. The colloidal size changes from around 7 nm to around 200 nm after adding 40% PEABr, which should be ascribed to the formation of PEABr clusters in DMSO. Since there is an interaction between the cluster and lead polyhalide, only one DLS peak is observed. With the addition of crown, the

cluster average size reduces to approximately 4 nm, which is interpreted by suppressing PEABr-induced aggregation. We thus speculate that PEA cations and lead polyhalide framework displays strong interaction in the form of a complex components with a large size. In order to verify this assumption, pristine PEABr solutions with and without crown in DMSO are also measured. As shown in Supplementary Figure 15b, pristine PEABr aggregation in DMSO is easily observed. As we expected, this aggregation disappeared with adding crown, which confirms PEABr self-crystallization.

Supplementary Note 5 - Temperature dependent PL measurements

Temperature dependence of the PL spectra is shown in Supplementary Figure 16a, b and c. All the samples show an asymmetric PL shape at low temperature, which reveals that sub-band-gap state radiative relaxation is likely to contribute PL emission besides band-to-band one[27-30]. A temperature dependent PL measurement for single crystal $CsPbBr_3$ by the Bridgman method shows one strong PL peak at 532 nm (2.33 eV) and weak one at approximately 540 nm (2.29 eV) at 10 K[29]. In addition, energy transfer from band-to-band to sub-band-gap state occurs[31]. Regarding to 0% PEABr OHP film, the PL shows slight blue PL shift with increasing temperature, and it displays a strong peak at 530 nm and weak red tail at 77 K, which is consistent with previous temperature dependent single crystal of $CsPbBr_3$ growth by the Bridgman method[29]. Recently, white light emission is observed in <001> orientated bromide 2D perovskite at low temperature[30]. The low-energy emission originates from sub-band-gap states instead of a new bulk phase[30]. A low-symmetry phase transition change results in the stacking of platelet sheets from eclipsed to staggered at low temperature. However, it is still unclear why the broad sub-band-gap emission in these <001> orientated bromide 2D perovskite only occurs at low temperature[30]. Here, regarding to 40% PEABr OHP film, low-energy emission becomes stronger with reducing temperature, which is likely to assign 2D bromide perovskite sub-band-gap state emission. With incorporating crown, dimensional distribution of 2D perovskite is changed, which dramatically effects sub-band-gap state emission in 40% PEABr-crown based OHP sample at low temperature. In addition, the band-to-band

emission becomes very weak because efficient energy transfers from band-to-band to sub-band-gap state emission when temperature is below 140 K[29]. This observation is also consistent with a fact that more asymmetric peak is observed with incorporation of crown even at room temperature, as shown in Figure 2b. The low-energy emission difference between perovskite with and without crown is correlated with their variation of 2D platelet dimensional distribution.

We have also analyzed the temperature dependence of the integrated PL signal in terms of a standard model that is used to roughly valuate exciton binding energy values[32]. The plot of temperature (T)-dependent PL intensity (I(T)) is shown in Supplementary Figure 16 (d), which can be fitted using:

$$I(\mathrm{T}) = \frac{I_0}{1 + Ae^{-E_\mathrm{b}/k_\mathrm{B}T}}$$

Here, I_0 is the PL intensity at 0 K, and k_B is the Boltzmann constant. According to the fitting results, E_b values are extracted of approximately 40.5$\pm$1.4 meV, 63.8$\pm$1.3 meV and 69.5$\pm$2.2 meV for pristine $CsPbBr_3$, 40% PEABr perovskite and 40% PEABr-crown perovskite films, respectively. Obviously, 40% PEABr-crown film displays the largest binding energy, which is qualitatively in line well with the strongest PL intensity and the notion of a more uniform crystallite environment and higher dielectric confinement.

Supplementary Note 6 - Discussion of exciton binding energy

After addition of crown, PLQY is dramatically enhanced, as shown in Figure 2c. This enhanced PLQY should be correlated with increased mono-molecular recombination ratio, which will be discussed later. We speculate that a larger exciton binding energy E_b is achieved with incorporation of PEABr compared to pristine $CsPbBr_3$. Consequently, increased E_b would result in a reduced optical bandgap (E_{opt}) as E_g is constant, as shown in Supplementary Figure 17. Thus, a red-shift in PL is observed with incorporation of crown.

Supplementary Note 7 - Alternative OHP based on different organic ligand with adding crown

We have found that the effect of PLQY enhancement upon crown addition can also be observed for OHP films based on different organic ligands. Supplementary Figure 18 shows XRD diffraction patterns of OHP films formed with phenylmethanamium bromide (PMABr) or BABr and Supplementary Table 2 shows the corresponding PLQY. Analogous to PEABr, the PMA based OHP film with crown yield PLQY of 21.6% due to suppressed PMABr crystallization, which is dramatically improved in comparison with that of the PMABr-only based OHP film. Also, for BABr a significant, though not quite as large increase in PLQY is observed. In this case we have no evidence for BABr crystallization from the XRD. The increased PL in BABr based OHP may reflect different size distributions of nanocrystals in the films affecting the efficiency of energy transfer. This will need to be investigated in more detail in future work.

Supplementary Note 8 - LED device characterization

In Supplementary Figure 19-25, we present more detailed LED characterization.

Supplementary Note 9 - LEDs based on colloidal $CsPbBr_3$ nanocrystal

It is worth noting that a state-art-of $CsPbBr_3$ nanocrystal can achieve PLQY of 100% via carefully tuning chelating ligand to minimize the surface defect[33]. It is expected that these $CsPbBr_3$ nanocrystal should be an excellent candidate for LED. However, because an insulating long chelating ligand layer (oleic acid and oleylamine) is always present for nanocrystal surface passivation and solution dispersing capability, charge transport becomes a great challenge in solid $CsPbBr_3$ nanocrystal film. Here, a high quality $CsPbBr_3$ nanocrystal with PLQY of approximately 92% is achieved (absorption, PL and transmission electron microscopy (TEM) images show in Supplementary Figure 26). The nanocrystal with diameter of approximately 10 nm is comparable with light emitting nanocrystal in crown-based sample (approximately 13 nm). However, a LED device with as similar film thickness (approximately 40 nm, 4 to 5 nanocrystals in vertical direction) as precursor based one only achieves a luminescence of 6 cd m^{-2}, as shown in Supplementary Figure 26b. A very high resistivity is observed due to the presence of an insulating long ligands covering $CsPbBr_3$ nanocrystal. If we reduce the nanocrystal film thickness to approximately 15 nm (2 to 3 nanocrystal in vertical direction), an EQE of approximately 5% is achieved, which is comparable with the present of state-of-art $CsPbBr_3$ nanocrystal based LED[34]. It confirms that the charge transport is still a limited factor for $CsPbBr_3$ nanocrystal based devices. However, the precursor based perovskite films consist of much shorter organic ligands, the transport is not limited by the ligand resistivity.

Supplementary Note 10 - Time-resolved PL and TA measurements

In Supplementary Figure 27-30, we present more detailed time-resolved PL and TA characterization.

Supplementary References

1. Tan, Z.-K. et al. Bright light-emitting diodes based on organometal halide perovskite. *Nat. Nanotechnol.* **9**, 687-692 (2014).
2. Kim, Y.-H. et al. Multicolored Organic/Inorganic Hybrid Perovskite Light-Emitting Diodes. *Adv. Mater.* **27**, 1248-1254 (2015).
3. Yuan, M. et al. Perovskite energy funnels for efficient light-emitting diodes. *Nat. Nanotechnol.* **11**, 872-877 (2016).
4. Chen, Z. et al. High-Performance Color-Tunable Perovskite Light Emitting Devices through Structural Modulation from Bulk to Layered Film. *Adv. Mater.* **29**, 1603157 (2017).
5. Wang, N. et al. Perovskite light-emitting diodes based on solution-processed self-organized multiple quantum wells. *Nat. Photon.* **10**, 699-704 (2016).
6. Xiao, Z. et al. Efficient perovskite light-emitting diodes featuring nanometre-sized crystallites. *Nat. Photon.* **11**, 108-115 (2017).
7. Zhang, S. et al. Efficient Red Perovskite Light-Emitting Diodes Based on Solution-Processed Multiple Quantum Wells. *Adv. Mater.* **29**, 1606600 (2017).
8. Quan, L.N. et al. Tailoring the Energy Landscape in Quasi-2D Halide Perovskites Enables Efficient Green-Light Emission. *Nano Lett.* **17**, 3701-3709 (2017).
9. Zhang, L. et al. Ultra-bright and highly efficient inorganic based perovskite light-emitting diodes. *Nat. Commun.* **8**, 15640 (2017).
10. Yang, X. et al. Efficient green light-emitting diodes based on quasi-two-dimensional composition and phase engineered perovskite with surface passivation. *Nat. Commun.* **9**, 570 (2018).
11. Yu, J.C. et al. High-Performance Planar Perovskite Optoelectronic Devices: A Morphological and Interfacial Control by Polar Solvent Treatment. *Adv. Mater.* **27**, 3492-3500 (2015).
12. Zhang, X. et al. Enhancing the Brightness of Cesium Lead Halide Perovskite Nanocrystal Based Green Light-Emitting Devices through the Interface Engineering with Perfluorinated Ionomer. *Nano Lett.* **16**, 1415-1420 (2016).
13. Wei, Z. et al. Solution-processed highly bright and durable cesium lead halide perovskite light-emitting diodes. *Nanoscale* **8**, 18021-18026 (2016).
14. Li, J. et al. Single-Layer Halide Perovskite Light-Emitting Diodes with Sub-Band Gap Turn-On Voltage and High Brightness. *J. Phys. Chem. Lett.* **7**, 4059-4066 (2016).
15. Shi, Z. et al. High-Efficiency and Air-Stable Perovskite Quantum Dots Light-Emitting Diodes with an All-Inorganic Heterostructure. *Nano Lett.* **17**, 313-321 (2017).
16. Xiao, Z. et al. Efficient perovskite light-emitting diodes featuring nanometre-sized crystallites. *Nat. Photon.* **11**, 108 (2017).
17. Lee, J.-W. et al. In-Situ Formed Type I Nanocrystalline Perovskite Film for Highly Efficient Light-Emitting Diode. *ACS Nano* **11**, 3311-3319 (2017).
18. Zhang, X. et al. Hybrid Perovskite Light-Emitting Diodes Based on Perovskite Nanocrystals with OrganicБ–⌠Inorganic Mixed Cations. *Adv. Mater.* **29**, 1606405 (2017).
19. Zhao, L. et al. Electrical Stress Influences the Efficiency of CH3NH3PbI3 Perovskite Light Emitting Devices. *Adv. Mater.* **29**, 1605317 (2017).
20. Wu, C. et al. Improved Performance and Stability of All-Inorganic Perovskite Light-Emitting

Diodes by Antisolvent Vapor Treatment. *Adv. Funct. Mater.* **27**, 1700338 (2017).

21. Zhang, L. et al. Ultra-bright and highly efficient inorganic based perovskite light-emitting diodes. *Nat. Commun.* **8**, 15640 (2017).

22. Tsai, H. et al. Stable Light-Emitting Diodes Using Phase-Pure RuddlesdenБ─⌈Popper Layered Perovskites. *Adv. Mater.* **30**, 1704217 (2018).

23. Zou, W. et al. Minimising efficiency roll-off in high-brightness perovskite light-emitting diodes. *Nat. Commun.* **9**, 608 (2018).

24. Mitzi, D.B. Templating and structural engineering in organic–inorganic perovskites. *J. Chem. Soc., Dalton Trans.* **0**, 1-12 (2001).

25. Tidhar, Y. et al. Crystallization of Methyl Ammonium Lead Halide Perovskites: Implications for Photovoltaic Applications. *J. Am. Chem. Soc.* **136**, 13249-13256 (2014).

26. McMeekin, D.P. et al. Crystallization Kinetics and Morphology Control of Formamidinium–Cesium Mixed-Cation Lead Mixed-Halide Perovskite via Tunability of the Colloidal Precursor Solution. *Adv. Mater.* **29**, 1607039 (2017).

27. Stoumpos, C.C. & Kanatzidis, M.G. The Renaissance of Halide Perovskites and Their Evolution as Emerging Semiconductors. *Acc. Chem. Res.* **48**, 2791-2802 (2015).

28. Kang, J. & Wang, L.-W. High Defect Tolerance in Lead Halide Perovskite CsPbBr3. *J. Phys. Chem. Lett.* **8**, 489-493 (2017).

29. Sebastian, M. et al. Excitonic emissions and above-band-gap luminescence in the single-crystal perovskite semiconductors $CsPbBr_3$ and $CsPbCl_3$. *Phys. Rev. B* **92**, 235210 (2015).

30. Booker, E.P. et al. Formation of Long-Lived Color Centers for Broadband Visible Light Emission in Low-Dimensional Layered Perovskites. *J. Am. Chem. Soc.* **139**, 18632-18639 (2017).

31. Nitsch, K., Hamplová, V., Nikl, M., Polák, K. & Rodová, M. Lead bromide and ternary alkali lead bromide single crystals — growth and emission properties. *Chem. Phys. Lett.* **258**, 518-522 (1996).

32. Wu, K. et al. Temperature-dependent excitonic photoluminescence of hybrid organometal halide perovskite films. *Phys. Chem. Chem. Phys.* **16**, 22476-22481 (2014).

33. Koscher, B.A., Swabeck, J.K., Bronstein, N.D. & Alivisatos, A.P. Essentially Trap-Free CsPbBr3 Colloidal Nanocrystals by Postsynthetic Thiocyanate Surface Treatment. *J. Am. Chem. Soc.* **139**, 6566-6569 (2017).

34. Li, J. et al. 50-Fold EQE Improvement up to 6.27% of Solution-Processed All-Inorganic Perovskite CsPbBr3 QLEDs via Surface Ligand Density Control. *Adv. Mater.* **29**, 1603885 (2017).
